# Dual-chamber injection device for measles-rubella vaccine: The potential impact of introducing varying sizes of the devices in 3 countries

**DOI:** 10.1016/j.vaccine.2018.08.026

**Published:** 2018-09-18

**Authors:** Patrick T. Wedlock, Elizabeth A. Mitgang, Sheryl S. Siegmund, Jay DePasse, Jennifer Bakal, Jim Leonard, Joel Welling, Shawn T. Brown, Bruce Y. Lee

**Affiliations:** a HERMES Logistics Modeling Team, Baltimore, MD and Pittsburgh, PA, USA; b Global Obesity Prevention Center (GOPC) at Johns Hopkins University, Johns Hopkins Bloomberg School of Public Health, Baltimore, MD, USA; c Pittsburgh Supercomputing Center, Carnegie Mellon University, Pittsburgh, PA, USA; d McGill Centre for Integrative Neuroscience, McGill Neurological Institute, McGill University, Montreal, Canada

**Keywords:** Dual-chamber injection device, Measles, Supply chain

## Abstract

**Introduction:**

By pairing diluent with vaccines, dual-chamber vaccine injection devices simplify the process of reconstituting vaccines before administration and thus decrease associated open vial wastage and adverse events. However, since these devices are larger than current vaccine vials for lyophilized vaccines, manufacturers need guidance as to how the size of these devices may affect vaccine distribution and delivery.

**Methods:**

Using HERMES-generated immunization supply chain models of Benin, Bihar (India), and Mozambique, we replace the routine 10-dose measles-rubella (MR) lyophilized vaccine with single-dose MR dual-chamber injection devices, ranging the volume-per-dose (5.2–26 cm^3^) and price-per-dose ($0.70, $1.40).

**Results:**

At a volume-per-dose of 5.2 cm^3^, a dual-chamber injection device results in similar vaccine availability, decreased open vial wastage (OVW), and similar total cost per dose administered as compared to baseline in moderately constrained supply chains. Between volumes of 7.5 cm^3^ and 26 cm^3^, these devices lead to a reduction in vaccine availability between 1% and 14% due to increases in cold chain storage utilization between 1% and 7% and increases in average peak transport utilization between 2% and 44%. At the highest volume-per-dose, 26 cm^3^, vaccine availability decreases between 9% and 14%. The total costs per dose administered varied between each scenario, as decreases in vaccine procurement costs were coupled with decreases in doses administered. However, introduction of a dual-chamber injection device only resulted in improved total cost per dose administered for Benin and Mozambique (at 5.2 cm^3^ and $0.70-per-dose) when the total number of doses administered changed <1% from baseline.

**Conclusion:**

In 3 different country supply chains, a single-dose MR dual-chamber injection device would need to be no larger than 5.2 cm^3^ to not significantly impair the flow of other vaccines.

## 1 Introduction

By pairing diluent with vaccines, dual-chamber vaccine injection devices [1], [2], [3] simplify the process of reconstituting vaccines before administration and thus decrease associated open vial wastage [4], [5], [6] and adverse events [7], [8], [9]. However, since these devices are larger than current vaccine vials for lyophilized vaccines, manufacturers need guidance as to how the size of these devices may affect vaccine distribution and delivery. Many routine supply chains are constrained [10], [11], and increasing the volume of vaccines in these systems can have multiple negative effects [12], [13]. At larger sizes, dual-chamber devices may offset the benefits of reduced wastage and fewer adverse events by increasing cold storage utilization and limiting the availability of other vaccines. In addition, this decrease in available storage space could impede the potential for new vaccine introductions. The earlier vaccine manufacturers can identify the device size above which a supply chain becomes more constrained and incorporate this into their technology, the more time and resources can be saved in creating a successful product [14], [15].

To identify the ideal size of dual-chamber injection devices for use in routine immunization programs, this study uses vaccine supply chain models of the Republic of Benin, Bihar (India), and the Republic of Mozambique, developed using the HERMES supply chain modeling software, to simulate the effects of replacing 10-dose lyophilized measles-rubella (MR) vaccines with single-dose dual-chamber injection devices of varying sizes and prices. The optimal volume-per-dose was identified as either improving or causing no change in vaccine availability, vaccine wastage, and supply chain costs compared to the existing program.

## 2 Methods

### 2.1 HERMES vaccine supply chain models: Benin, Bihar, Mozambique

Our team used three previously developed stochastic, discrete-event vaccine supply chain simulation models of Benin [16], [17], [18], Bihar [19] and Mozambique [20]. These models were developed using the Highly Extensible Resource for Modeling Supply Chains (HERMES) software and virtually represent all of the storage facilities, refrigerators and freezers, shipping routes, vehicles, personnel, and vaccines in each supply chain.

For all fixed parameters, including cold storage and transport storage capacity, travel times on each route, personnel at each facility, and associated unit costs for each of these, data was provided from country partners for each model. To better simulate the reality of each supply chain, certain parameters that are not static, like the number of vaccination sessions per day at a given clinic, are stochastically drawn from distributions (e.g. a Poisson distribution of the average number of vaccinations per session).

The Bihar model is based on data from 2013 to 2014 and consists of four levels, including one state store, seven division-level stores, 38 district stores, and 425 primary health centers (PHCs) [21]. The birth cohort population modeled is 2,997,442. The model for Benin is based on data from 2012 and consists of one national store, six departmental stores and one regional store, 80 commune stores, and 763 service delivery points [16], [17], [18]. The birth cohort population modeled is 371,022. The Mozambique model is based on data from 2014 and consists of one national store (which doubles as a provincial store), 10 strictly provincial stores, 104 district stores, 1428 health facilities and 254 mobile brigades. The birth cohort population modeled is 1,085,363 [20].

### 2.2 Comparing 10-dose MR vials to single-dose MR dual-chamber injection devices

In order to simulate the effects of replacing the current 10-dose MR vials with single-dose MR dual chamber injection devices, we included 10-dose MR vials in the standard EPI (Expanded Programme on Immunization) schedules of Benin, Bihar, and Mozambique, replacing the measles (M) vaccine. We maintained all other vaccines that were included in the routine immunization schedules in the years for which data was provided to create the models (see Section 2.1). Table 1 includes the EPI vaccines modeled in each supply chain. The 10-dose lyophilized MR presentation has a vaccine volume-per-dose of 2.1 cm^3^ and a diluent volume-per-dose of 3.1 cm^3^ for a total volume of 5.2 cm^3^. Diluent provided with the MR vaccine can be stored at room temperature through the majority of the supply chain, being placed into refrigeration only prior to reconstitution at the service delivery level. By contrast, dual-chamber injection devices are designed to include both the vaccine and the diluent in the same unit, therefore requiring the diluent to be stored alongside the vaccine throughout the entire cold chain. We varied the volume-per-dose of the single-dose MR dual-chamber injection device from 5.2 cm^3^, representing the current total volume of 10-dose lyophilized MR vaccine plus diluent, to 26 cm^3^, which is the current total volume-per-dose of single-dose lyophilized MR and diluent.

**Table 1:** Vaccine characteristics.

Supply chain model	Vaccine	Doses per vial	Packed vol (cm^3^)/dose of vaccine (*combined vaccine & diluent for MR DCID*)	Packed vol (cm^3^)/dose of diluent	Price per vial
Benin	BCG	20	1.2	0.7	$ 2.16
	Measles-Rubella/MR DCID^a^	10/1	2.1/5.2–26	3.1/diluent incl. in vol-per-dose of vaccine	$ 6.30/$ 0.70, $1.40
	OPV	20	1	0	$ 2.40
	PCV13	1	12	0	$ 3.30
	Pentavalent	2	11	0	$ 3.88
	Tetanus Toxoid	10	3	0	$ 6.10
	Yellow Fever	10	2.5	6	$ 1.10

Bihar	BCG	20	1.2	0.7	$ 2.16
	DTP	10	3	0	$ 1.78
	Hepatitis B	10	3.8	0	$ 2.10
	Japanese Encephalitis	5	3	2.9	$ 2.10
	Measles-Rubella/MR DCID	10/1	2.1/5.2–26	3.1/diluent incl. in vol-per-dose of vaccine	$ 4.90/$ 0.70, $1.40
	OPV	20	1	0	$ 2.40
	Tetanus Toxoid	10	3	0	$ 6.10

Mozambique	BCG	20	1.2	0.7	$ 2.16
	IPV	1	14.3	0	$ 2.80
	Measles-Rubella/MR DCID	10/1	2.1/5.2–26	3.1/diluent incl. in vol-per-dose of vaccine	$ 6.30/$ 0.70, $1.40
	OPV	10	2	0	$ 1.25
	PCV10	2	4.8	0	$ 6.10
	Pentavalent	10	2.6	0	$ 6.90
	Rotavirus	1	17.1	0	$ 2.27
	Tetanus Toxoid	10	2.61	0	$ 1.29

a MR DCID: Measles-rubella dual-chamber injection device.

#### 2.2.1 Vaccine procurement in HERMES

The HERMES model procures vaccines “fairly” when storage capacity is constrained. The model divides the total volume of all vaccines needing to be stored in a given storage type (e.g. refrigerator) by the total space available to calculate a percentage, which is then applied equally to the total volumes needed for each vaccine. If the total volume needed for a certain vaccine increases (e.g. due to an increase in volume-per-dose), then the volume of that vaccine procured will increase as well, reducing storage space for other vaccines.

### 2.3 Experimental scenarios

For each model we compared the presentation of standard 10-dose lyophilized MR vaccine to the impact of replacing 10-dose lyophilized MR vaccines with single-dose MR dual-chamber injection devices at sizes of 5.2 cm^3^, 7.5 cm^3^, 10 cm^3^, 15.2 cm^3^, and 26 cm^3^. Each simulation involved running the model for a period of one year. Each experiment was averaged over 23 runs and the results had a standard deviation within 1% of the mean. In order to capture a range of potential price points for the single-dose MR dual-chamber injection device, we included cost results at both $0.70-per-dose and $1.40-per-dose based on price estimates provided by expert opinion.

## 3 Results

### 3.1 Current situation with 10-dose MR vials in Benin, Bihar, and Mozambique’s supply chains

Table 2 includes baseline results for vaccine availability (i.e. successful immunizations administered to patients as % of immunizations needed), open vial wastage, and the costs per dose administered for each supply chain. In Benin, using current vaccine presentations, 4,622,097 doses of all vaccines were administered and 798,065 doses of all vaccines were wasted. In Bihar, using current vaccine presentations, 15,237,561 doses of all vaccines were administered and 2,842,314 doses of all vaccines were wasted. And in Mozambique, using current vaccine presentations, 13,472,989 doses of all vaccines were administered and 4,268,842 doses of all vaccines were wasted.

**Table 2:** Baseline results for Benin, Bihar, and Mozambique’s routine vaccine supply chains.

Supply chain model	Vaccine availability		Open vial wastage		Cost per dose administered (in 2018 $US)	
	*MR*	*Total*	*MR*	*Total*	*Logistics*	*Total*
Benin	82%	90%	47%	15%	$ 0.21	$ 1.53
Bihar	61%	64%	49%	16%	$ 0.04	$ 1.01
Mozambique	66%	67%	47%	24%	$ 0.33	$ 1.90

The average peak transport utilization (i.e. the maximum percentage of available transport capacity needed to complete any shipment averaged over all routes) in Benin, Bihar, and Mozambique was 104%, 95%, and 199%, respectively. In Benin, the average peak storage utilization (i.e. the maximum percentage of available storage capacity occupied by products at any time, averaged by supply chain level) was 98% at the central level, 39% at the department level, 39% at the commune level and 8% at the health post level. In Bihar, the corresponding efficiency measure was 92% at the state level, 27% at the district level, and 17% at the PHC level, and in Mozambique this was 99% at the national level, 61% at the provincial level, 66% at the district level, and 29% at the health center level.

The total logistics costs for each supply chain were $961,417 in Benin, $579,713 in Bihar, and $4,419,198 in Mozambique, while the total vaccine procurement costs were $6,110,044, $14,793,148, and $21,166,287, respectively.

### 3.2 Effects on vaccine availability and doses administered of replacing 10-dose MR vials with single-dose dual-chamber injection devices

**Fig. 1:**
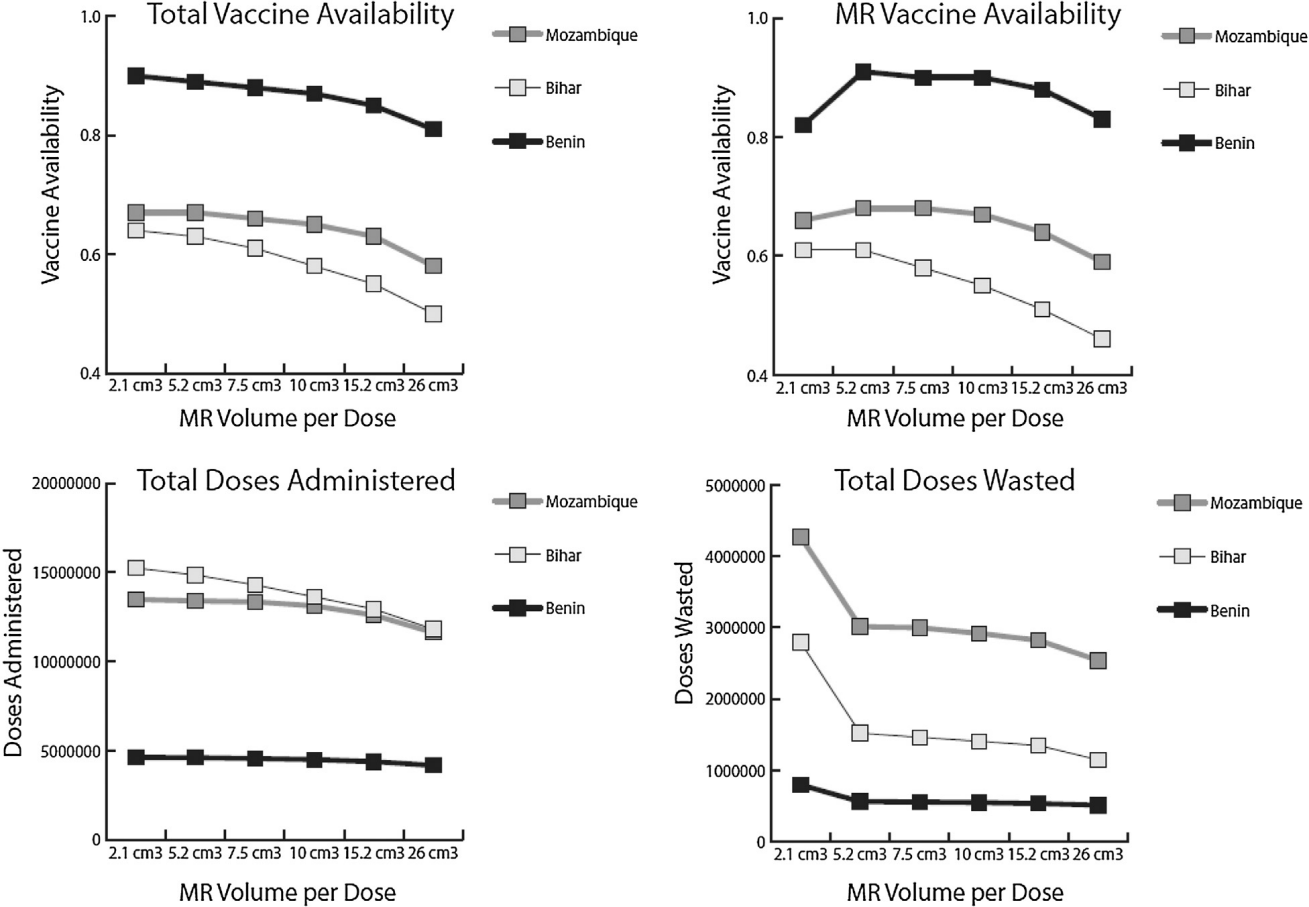
Vaccine availability, doses administered, and doses wasted by MR volume-per-dose and supply chain. *2.1 cm^3^ represents the use of the 10-dose MR vial, while 5.2 cm^3^ to 26 cm^3^ represents the use of a dual-chamber injection device.

Fig. 1 presents total vaccine availability, MR vaccine availability, doses administered, and doses wasted by MR volume-per-dose for each supply chain.

Replacing 10-dose MR vials with single-dose MR dual-chamber injection devices had positive and negative effects on vaccine availability and the number of doses both administered and wasted, driven primarily by changes in open vial wastage and changes in total vaccine volume, which exacerbated existing cold storage constraints (explained in more detail in Section 3.3).

Introducing single-dose devices had a positive effect on MR and total open vial wastage. The single-dose MR presentation meant that no doses of MR vaccine would be wasted. This saved 322,752 MR doses in Benin, 1,277,320 MR doses in Bihar, and 1,266,683 MR doses in Mozambique, reducing total OVW to 11%, 9%, and 18%, respectively. As fewer doses of MR vaccine were being wasted, MR vaccine availability increased in Benin at all dual-chamber device volumes and in Mozambique at volumes-per-dose of 10 cm^3^ or less. MR vaccine availability did not increase in Bihar even as MR OVW went to 0%. While many MR doses were saved, the supply chain is unable to order and stock enough dual-chamber injection devices to meet demand due to cold storage and transport constraints.

While open vial wastage decreased in each supply chain and MR availability increased in some scenarios, the total number of doses administered and the total vaccine availability gradually decreased in each scenario as the volume-per-dose increased. These supply chains are constrained, and introducing a larger volume-per-dose MR vaccine resulted in a greater percentage of cold chain storage dedicated to MR injection devices (which contain both the vaccine and diluent). This reduced the available cold storage space for other vaccines, resulting in fewer non-MR vaccines procured, and reducing the availability of these vaccines.

### 3.3 Effects on cold chain utilization of replacing 10-dose MR vials with single-dose dual-chamber injection devices

**Fig. 2:**
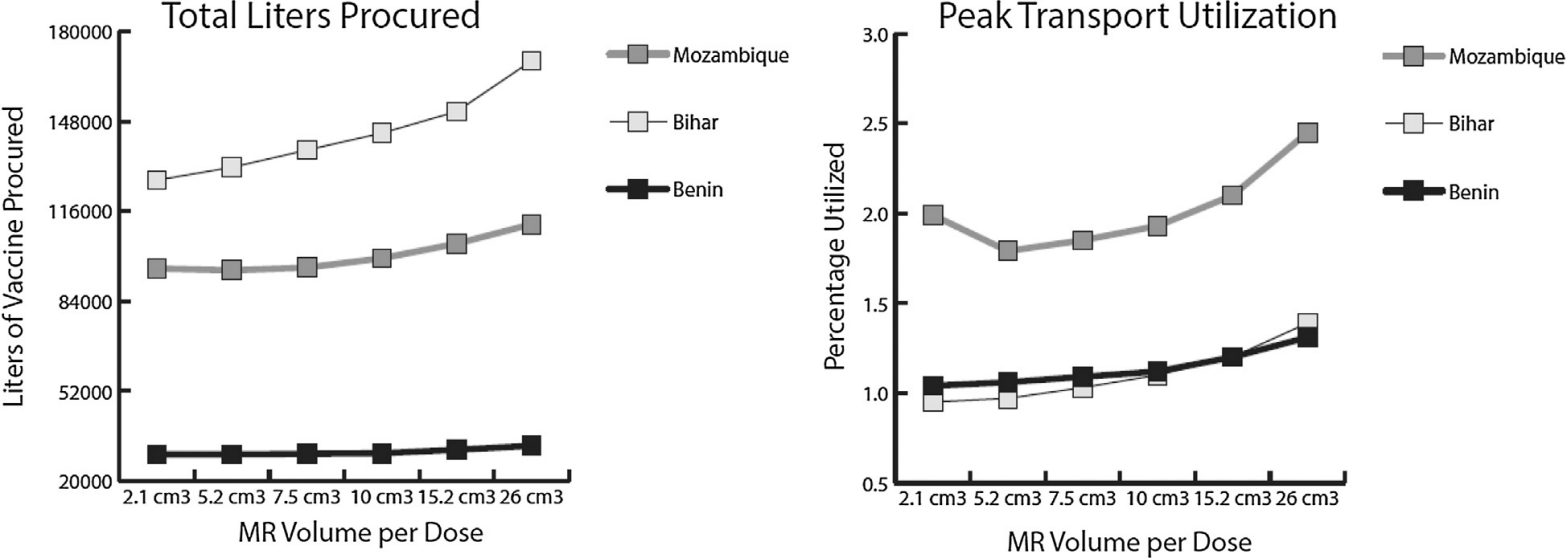
Vaccine (L) procured and peak transport utilization by MR volume-per-dose and supply chain. *2.1 cm^3^ represents the use of the 10-dose MR vial, while 5.2 cm^3^ to 26 cm^3^ represents the use of a dual-chamber injection device.

Fig. 2 presents the total liters of vaccine procured and average peak transport utilization by MR volume-per-dose for each supply chain.

Each supply chain modeled had existing storage and transport constraints at baseline. The top-level storage location in each supply chain was highly utilized (between 92% and 99%) and each supply chain had multiple routes requiring more than the maximum transport capacity for each shipment. Switching from 10-dose MR vials with a volume-per-dose of 2.1 cm^3^ to single-dose MR dual-chamber injection devices increased the MR volume-per-dose between 2.5× and 12×. While fewer total doses of MR needed to be procured (due to the reduction in open vial wastage), this did not offset the increase in total MR volume needed due to the increase in MR volume-per-dose. As such, even at 5.2 cm^3^, the supply chains needed to order a larger total volume of MR just to match baseline MR availability.

Switching to dual-chamber injection devices of any size increased top-level storage constraints to 99% in each supply chain, and nearly always led to an increase in the average peak transport utilization. This increase in cold chain utilization by MR resulted in fewer doses of all non-MR vaccines entering and moving through the system, subsequently reducing total doses administered and total vaccine availability.

### 3.4 Effects on supply chain costs of replacing 10-dose MR vials with single-dose dual-chamber injection devices

**Fig. 3:**
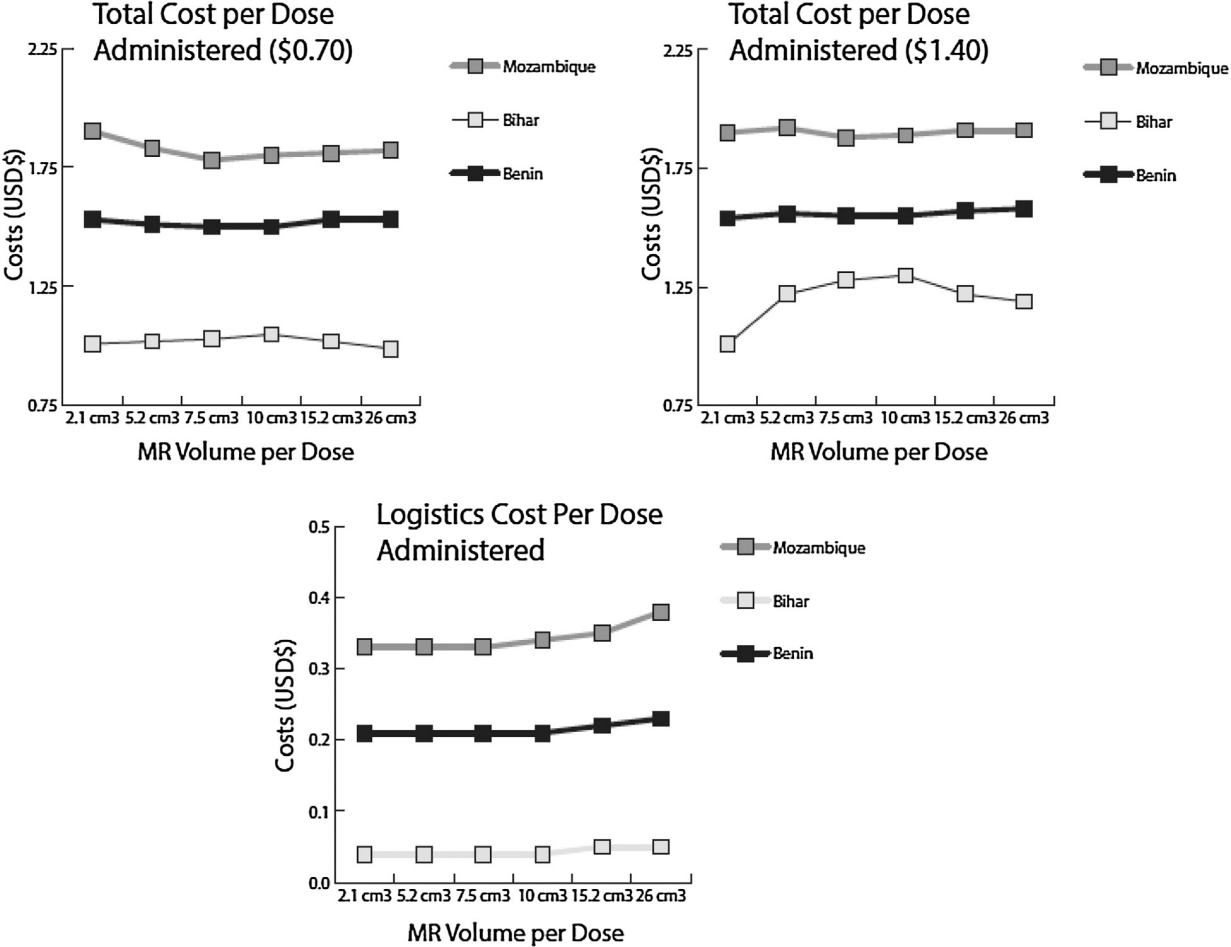
Logistics cost and total cost per dose administered by MR volume-per-dose and supply chain. *2.1 cm^3^ represents the use of the 10-dose MR vial, while 5.2 cm^3^ to 26 cm^3^ represents the use of a dual-chamber injection device.

Fig. 3 presents the logistics cost per dose administered and total cost per dose administered by MR volume-per-dose for each supply chain.

Introducing the dual-chamber injection devices had various effects on logistics costs and vaccine procurement costs. Logistics costs (comprised of the costs of storage equipment, buildings, vehicles, and labor) generally decreased in Mozambique with the introduction of the single-dose MR dual-chamber injection devices. This decrease was due to fewer doses wasted, and fewer trips needed to procure additional vaccines. Since storage capacity remained the same, the costs of storage equipment, buildings, and labor generally did not change from baseline. In Benin, logistics costs remained approximately the same compared to baseline. In Bihar, however, logistics costs increased with the introduction of MR injection devices as vehicles there could “cope” with the increase in volume by taking multiple additional trips as needed. The increase in trips led to an increase in associated transport costs.

These devices also had both positive and negative effects on vaccine procurement costs. At $0.70-per-dose and $1.40-per-dose, total vaccine procurement costs decreased in both Benin and Mozambique. Bihar, which had a lower initial price-per-dose of MR vaccine, saw an increase in vaccine procurement costs at both injection device price points. Two mechanisms helped reduce vaccine procurement costs in Benin and Mozambique: (1) the almost 50% reduction in MR doses needed and the near-equal price-per-dose of MR injection devices meant that the total price of procuring MR vaccines decreased, and (2) the decrease in cold chain space available for non-MR vaccines meant that fewer non-MR vaccines could be procured, leading to a reduction in total non-MR vaccine procurement costs. The first mechanism is a positive effect of switching to an affordable single-dose MR dual-chamber injection device. The second mechanism may seem to be positive for reducing costs, but this is driven by a decrease in total vaccine availability. To maintain vaccine availability, additional storage, transport, or other logistics costs would be incurred, driving up the total cost per dose administered.

When total doses administered are factored in, the logistics cost per dose and the total cost per dose seem to indicate a benefit compared to baseline. However, at each MR injection device volume-per-dose, the total number of doses administered decreases from baseline. This means that even though there may be positive effects on logistics costs and vaccine procurement costs with the switch to MR injection devices, the logistics cost per dose administered and total cost per dose administered fluctuate due to the combination of both the positive and negative effects of the vaccine.

## 4 Discussion

Based on these results, a dual-chamber injection device for lyophilized MR vaccine that is larger than 5.2 cm^3^ (approximately the combined volume-per-dose of vaccine and diluent for a 10-dose MR vaccine) would exacerbate existing bottlenecks in moderately constrained supply chains. Without adjusting supply chain capacity to accommodate the increase in volume, dual-chamber devices above 5.2 cm^3^ lead to a reduction in total vaccine availability and opportunities to introduce new vaccines.

The primary benefit of single-dose dual-chamber injection devices on routine immunization supply chains is reduced open vial wastage. When 10-dose MR vials were used, nearly half of all MR doses were wasted. Switching to a single-dose MR injection device reduced MR open vial wastage to 0 and meant that far fewer doses of MR vaccine needed to be procured. At a volume-per-dose of 5.2 cm^3^ in Benin and Mozambique, the dual-chamber injection device reduced open vial wastage by nearly 50%, while maintaining a total MR cold chain volume similar to baseline. When these dual-chamber MR vaccines were priced at $0.70 per dose, the total cost per dose administered improved or remained approximately the same. However, when the volume-per-dose increased above 5.2 cm^3^, the increase in volume of MR vaccine procured began to outweigh the decrease in volume due to preventing open vial wastage. As higher volumes of MR vaccine were procured in order to meet demand, the flow of all other routine vaccines diminished, resulting in a decrease in total vaccine availability.

Dual-chamber injection devices above 5.2 cm^3^-per-dose increased the volume of MR vaccine above what the existing cold chain capacity could handle, without a reduction in the procurement of other vaccines or adjustments to increase capacity. Each of the supply chains modeled had pre-existing cold storage constraints – a common issue in many low-resource vaccine supply chains [10], [11] – particularly in refrigeration at the top level and in transport storage. Even though fewer doses of MR needed to be procured, the increased size of the new devices was simply too large for the supply chains to handle. In an unconstrained supply chain system, with cold storage space and transport space available to meet increased volumes of MR vaccine, the introduction of a single-dose MR dual-chamber injection device would lead to an increase in total doses administered and total vaccine availability, reduce open vial wastage, and reduce total costs. However, without changes to the supply chains reflected in the model, identifying an unconstrained supply chain system is unlikely.

Overall, above a volume-per-dose of 5.2 cm^3^, the negative effects on the supply chain of broadly replacing 10-dose MR vials with MR dual-chamber injection devices outweigh the benefits. Manufacturers developing dual-chamber injection devices for lyophilized vaccines above this volume will need to consider the potential use-cases of their product, as broadly replacing 10-dose MR vials with larger single-dose dual-chamber devices in low- and middle-income country (LMIC) immunization programs will likely exacerbate cold chain constraints. These devices, which would reduce the incidence of adverse events from reconstituting vaccines, may provide more benefit to outreach or door-to-door campaigns where reconstitution of vaccines is more difficult. However, the benefits of reduced adverse events would likely not be enough to justify the subsequent reduction in availability of other vaccines that would occur when using dual-chamber injection devices in the routine immunization program.

## 5 Limitations

The three vaccine supply chains simulated in this study are models that represent real life but cannot fully capture every aspect of these complex systems. Each model maintains the supply chain storage and transport capacity (as it is input) over the course of the entire simulation, while, in reality, supply chains may incorporate additional cold storage or transport capacity when a location or route becomes too constrained. Because the MR dual-chamber injection device has not yet come to market, our model relies on data assumptions such as pricing characteristics informed by expert opinion, which is subject to change with additional experimentation and subsequent product development. In addition, due to this lack of market data, our outputs do not include information regarding physical wastage such as packaging. Our study uses models populated with supply chain and population data from 2012 to 2014, which does not capture the most recent state of each supply chain and demand.

Furthermore, the model does not capture the full potential safety benefits of dual-chamber injection devices. Such devices would have the potential to decrease unsafe needle reuse and to minimize the risk of improperly reconstituting the MR vaccine, and in turn, ineffective immunization. The modeled scenarios presented in this study only capture the effects of MR dual-chamber injection devices on routine immunization programs, and therefore do not capture the potential benefits of dual-chamber devices in outreach campaigns or door-to-door campaigns, where these devices might provide more of a comparative benefit.

## 6 Conclusion

In 3 different country supply chains, a single-dose MR dual-chamber injection device would need to be no larger than 5.2 cm^3^ to not significantly impair the flow of other vaccines without increasing cold chain capacity. Single-dose MR dual-chamber injection devices reduce open vial wastage at any volume and can lead to an increase in MR vaccine availability. However, the increase in volume-per-dose compared to the current 10-dose MR vials means that moderately constrained supply chains would be unable to handle the excess volume, resulting in fewer doses procured and administered.

## References

[1] Ingle R.G., Agarwal A.S. (2014). Pre-filled syringe – a ready-to-use drug delivery system: a review. Expert Opin Drug Deliv.

[2] Lloyd J.S. (2000). The technologies for vaccine delivery in the 21st century.

[3] Werk T., Ludwig I.S., Luemkemann J., Mahler H.C., Huwyler J., Hafner M. (2016). Technology, applications, and process challenges of dual chamber systems. J Pharm Sci.

[4] Yang W., Parisi M., Lahue B.J., Uddin M.J., Bishai D. (2014). The budget impact of controlling wastage with smaller vials: a data driven model of session sizes in Bangladesh, India (Uttar Pradesh), Mozambique, and Uganda. Vaccine.

[5] Parmar D., Baruwa E.M., Zuber P., Kone S. (2010). Impact of wastage on single and multi-dose vaccine vials: implications for introducing pneumococcal vaccines in developing countries. Hum Vaccin.

[6] Lee B.Y., Norman B.A., Assi T.-M. (2010). Single versus multi-dose vaccine vials: an economic computational model. Vaccine.

[7] Sood D.K., Kumar S., Singh S., Sokhey J. (1995). Adverse reactions after measles vaccination in India. Natl Med J India.

[8] Unicef. Building trust and responding to adverse events following immunization in south Asia: using strategic communication. Retrieved February. 2005;10:2016.

[9] Jodar L., Duclos P., Milstien J.B., Griffiths E., Aguado M.T., Clements C.J. (2001). Ensuring vaccine safety in immunization programmes — a WHO perspective. Vaccine.

[10] Ashok A., Brison M., LeTallec Y. (2017). Improving cold chain systems: challenges and solutions. Vaccine.

[11] Kaufmann J.R., Miller R., Cheyne J. (2011). Vaccine supply chains need to be better funded and strengthened, or lives will be at risk. Health Affairs.

[12] Haidari L.A., Wahl B., Brown S.T. (2015). One size does not fit all: the impact of primary vaccine container size on vaccine distribution and delivery. Vaccine.

[13] Assi T.-M., Brown S.T., Djibo A. (2011). Impact of changing the measles vaccine vial size on Niger's vaccine supply chain: a computational model. BMC Public Health.

[14] Lee B.Y., Burke D.S. (2010). Constructing target product profiles (TPPs) to help vaccines overcome post-approval obstacles. Vaccine.

[15] Duijzer L.E., van Jaarsveld W., Dekker R. (2018). Literature review: the vaccine supply chain. Eur J Oper Res.

[16] Brown S.T., Lee B.Y. (2014). Unless changes are made in Benin, multiple storage and transport bottlenecks may prevent vaccines from reaching the population. Vaccine.

[17] Brown S.T., Schreiber B., Cakouros B.E. (2014). The benefits of redesigning Benin's vaccine supply chain. Vaccine.

[18] Lee B.Y., Schreiber B., Wateska A.R. (2015). The Benin experience: how computational modeling can assist major vaccine policy changes in low and middle income countries. Vaccine.

[19] Lee B.Y., Bartsch S.M., Mui Y., Haidari L.A., Spiker M.L., Gittelsohn J. (2017). A systems approach to obesity. Nutr Rev.

[20] Lee B.Y., Haidari L.A., Prosser W. (2016). Re-designing the Mozambique vaccine supply chain to improve access to vaccines. Vaccine.

[21] Lee B.Y., Wedlock P.T., Haidari L.A. (2017). Economic impact of thermostable vaccines. Vaccine.

